# Increased CSF tau level is correlated with decreased lamina cribrosa thickness

**DOI:** 10.1186/s13195-015-0169-3

**Published:** 2016-02-08

**Authors:** Eun Ji Lee, Tae-Woo Kim, Dae Seung Lee, Hyunjoong Kim, Young Ho Park, Jungeun Kim, Joon Woo Lee, SangYun Kim

**Affiliations:** Department of Ophthalmology, Seoul National University Bundang Hospital and Seoul National University College of Medicine, 82, Gumi-ro, 173 Beon-gil, Bundang-gu, 463-707 Seongnam, Gyeonggi-do South Korea; Department of Applied Statistics, Yonsei University, 50 Yonsei-ro, Seodaemun-gu, 120-749 Seoul, South Korea; Department of Neurology, Seoul National University Bundang Hospital and Seoul National University College of Medicine, 82, Gumi-ro, 173 Beon-gil, Bundang-gu, 463-707 Seongnam, Gyeonggi-do South Korea; Department of Radiology, Seoul National University Bundang Hospital and Seoul National University College of Medicine, 82, Gumi-ro, 173 Beon-gil, Bundang-gu, 463-707 Seongnam, Gyeonggi-do South Korea

**Keywords:** Tau, Neurodegeneration, Optic nerve head, Lamina cribrosa, Spectral-domain optical coherence tomography

## Abstract

**Background:**

This study was to investigate whether the previously proposed link between Alzheimer’s disease (AD) and decreased retinal nerve fiber layer thickness could be explained by the relationship between abnormal CSF profiles and optic nerve head characteristics, focusing on the influence of CSF tau protein on the lamina cribrosa (LC) thickness (LCT).

**Methods:**

A total of 44 eyes from 18 patients with AD and 26 healthy subjects were subjected to enhanced-depth-imaging volume scanning of the optic nerve using spectral-domain optical coherence tomography. The B-scan images were constructed three-dimensionally using maximum intensity projection (MIP), and the LCT was measured at three locations (superior midperipheral, midhorizontal, and inferior midperipheral) using the thin-slab MIP images. CSF levels of amyloid β 1**-**42 peptide, (Aβ_1–42_), total tau (T-tau) and tau phosphorylated at threonine 181 (P-tau_181P_) were measured from CSF samples of each subject. The relationship between the level of CSF proteins and the LCT was determined using linear regression and fractional polynomial analyses.

**Results:**

Univariate regression analysis revealed that higher CSF levels of T-tau (*P* = 0.004) and P-tau_181P_ (*P* = 0.027), as well as a smaller central corneal thickness (*P* = 0.032), were significantly associated with a smaller LCT. Multivariate analysis indicated that only CSF T-tau (*P* = 0.041) was significantly associated with the LCT. The relationship was well explained by both linear regression (R^2^ = 0.179, *P* = 0.004) and fractional polynomial analysis (R^2^ = 0.275, *P* = 0.001). When we performed an assessment by linear regression with an indicator, the relationship was significant both in the healthy and AD groups, with a stronger correlation found in the healthy group (regression coefficients = -1.098 *vs.* -0.280, *P* = 0.018).

**Conclusions:**

An increased CSF level of T-tau was significantly associated with a thinner LCT in both the healthy and AD groups. This result suggests that LCT could serve as a potential non-invasive indicator for increased CSF tau. The clinical meaning of the higher level of CSF T-tau in axonal degeneration of the optic nerve remains to be determined.

## Background

Tau is a microtubule-associated protein that is most abundantly found in neurons and astrocytes within the central nervous system. Tau is known to play a pivotal role in various neurological pathways, interacting with signaling pathways that regulate microtubule stability and axonal transport [1–3]. Dysregulation of tau may disrupt microtubules, interfere with axon transport mechanisms, and be toxic to neurons; thus, the role of tau is central to many human neurodegenerative diseases, such as Alzheimer’s disease (AD), which are collectively referred to as tauopathies [1, 3–5]. An increased level of tau has also been suggested to be an important biomarker for AD [6].

The optic nerve is a part of the central nervous system, and mainly comprises retinal ganglion cell (RGC) axons that project to the optic chiasm and optic tract and ultimately form synapses at the lateral geniculate nucleus. Therefore, the optic nerve could be affected by similar degenerative processes during neurodegeneration. It has been suggested that pathogenic mechanisms similar to those in neurodegenerative diseases may also occur in retinal degenerative diseases such as glaucoma [5, 7–13].

Studies have suggested a possible role of tau protein in glaucomatous optic nerve damage. Yoneda et al. [14] demonstrated an increased level of vitreous tau in patients with retinal disease or those with glaucoma. Gupta et al. [15] showed that abnormal tau protein was present in the human retina with uncontrolled glaucoma. Experimental studies have also suggested that abnormal tau or tau inclusion in mouse RGCs is associated with reduced axonal transport [16], increased excitotoxic RGC degeneration [16], and reduced axonal outgrowth [17], However, the link between the tau protein and the RGC damage remains to be determined.

Glaucoma is a progressive optic neuropathy characterized by the degeneration of RGC axons. Currently, the lamina cribrosa (LC) within the optic nerve head (ONH) is considered the primary site of axonal damage in glaucoma [18–21]. It has been suggested that structural changes of the LC may induce damage of the RGC axons passing through the LC by various mechanisms [21, 22]. On the other hand, the structural differences of the LC are thought to be responsible for different effects of intraocular pressure (IOP) on the RGC axons within the ONH [21, 22]. It has been reported that the LC is thinner in glaucomatous eyes than in healthy eyes [23, 24]. Further, eyes with a thinner LC have also been associated with a worse glaucoma stage [23, 24] and a faster progression [25].

Considering the anatomical proximity between the ONH and retrobulbar CSF space, it is possible that the CSF protein affects the ONH. Given the implication of the LC in glaucoma pathogenesis and the possible influence of tau protein in glaucomatous axonal damage, it would be of interest to know how CSF tau protein is associated with the structural characteristics of the LC. The purpose of the present study was to determine the influence of CSF proteins on the ONH structure, focusing on the relationship between CSF tau and the lamina cribrosa thickness (LCT).

## Methods

This study is based on the Study for the Usefulness and Standardization of Cerebrospinal Fluid and Plasma Amyloid Biomarkers in Alzheimer’s Disease, which is an ongoing prospective study of patients with AD and healthy participants at the Neurocognitive Behavior Center in collaboration with the Glaucoma Clinic of Seoul National University Bundang Hospital. This study was approved by the Seoul National University Bundang Hospital Institutional Review Board. Informed written consent was obtained from all participants or their legally acceptable representatives in accordance with the Declaration of Helsinki.

### Study subjects

The subjects were enrolled by advertisement between May 2012 and April 2014. The participants included in the study were either normal or had been diagnosed with AD based on the National Institute on Aging–Alzheimer’s Association classification system [26].

Complete ophthalmic examinations were provided to all subjects, including a visual acuity assessment, Goldmann applanation tonometry, a refraction test, slit-lamp biomicroscopy, gonioscopy, a dilated stereoscopic examination of the optic disc, fundus photography (Kowa VX-10 fundus camera; Kowa Company, Tokyo, Japan), central corneal thickness measurements (Orbscan II; Bausch & Lomb Surgical, Rochester, NY, USA), corneal curvature measurements (KR-1800; Topcon, Tokyo, Japan), axial length measurements (IOL Master; Carl Zeiss Meditec, Dublin, CA, USA), and enhanced depth imaging (EDI) spectral-domain optical coherence tomography (SD-OCT) (Spectralis OCT; Heidelberg Engineering, Heidelberg, Germany) scanning of the optic disc and circumpapillary retinal nerve fiber layer (RNFL). The IOP was measured three times at 15-minute intervals, and the average of the three values was recorded as the baseline IOP. The subjects underwent cerebrospinal fluid (CSF) sampling with CSF pressure measurement on the same day as the ophthalmic examination (see ‘CSF sampling and storage’ section)*.*

To be included in the present study, an eye had to have a best-corrected visual acuity ≥20/40 with a spherical refraction of –8.0 to +5.0 diopters (D), cylinder correction within ±3.0 D, and a normal appearing optic disc without glaucomatous optic neuropathy and pallor or swelling. Eyes with any ocular diseases other than cataracts or a history of intraocular surgery other than cataract extraction were excluded. Subjects with a neurological disease other than AD, history of intracranial surgery, lumbar puncture prior to enrollment in this study, a condition that could affect the intracranial pressure (idiopathic intracranial hypotension/hypertension, intracranial tumors or medications (i.e., tetracycline, rofecoxib, mannitol, carbonic anhydrase inhibitor, etc.)), contraindication for lumbar puncture (i.e., infectious diseases or infection around the lumbar puncture site, elongated prothrombin time (International Normalized Ratio >1.7), thrombocytopenia <50,000/mm^3^, uremia, hemophilia, or other impairment of the coagulation process), or severe cognitive impairment that resulted in low cooperability were also excluded. When both eyes were eligible, one eye was randomly selected from each subject.

### EDI SD-OCT of the ONH

The ONH was imaged using Spectralis OCT with the EDI technique. The details of the protocol for scanning the optic nerve using EDI SD-OCT to evaluate the LC are described elsewhere [27, 28]. In brief, approximately 75 horizontal and vertical B-scan images covering the optic disc, 30–34 μm apart (the scan-line distance was determined automatically by the instrument), were obtained for each eye. For each section, 42 OCT frames were averaged, which provided the best trade-off between image quality and patient cooperation [28].

### Measurement of LCT

**Fig. 1:**
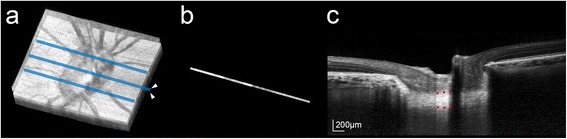
Measurement of the LCT. **a** MIP volumetric image reconstructed from B-scan images obtained using SD-OCT. *Light-blue lines* indicate the locations where the thin volumetric sections were obtained. **b** Thin-slab MIP image obtained at the center of the ONH (*blue line indicated with arrowheads* in **a**). **c** X–Y view of the thin-slab MIP image. The anterior and posterior borders of the LC were defined as the plane that best represented the margin of the hyper-reflective plate within the 200 μm wide area near the center of the Bruch’s membrane opening. The LCT was determined by measuring the distance between the anterior and posterior borders of the LC at three locations (*red glyphs*)

The LCT was measured at three locations in each eye (the midhorizontal and the superior and inferior midperipheral regions of the ONH) using thin-slab maximum intensity projection (MIP) images. Thin-slab MIP images were used because they allow a straighter detection of the posterior LC border [29]. The technique of generating thin-slab MIP images is described in detail elsewhere [29]. In brief, three-dimensional volumetric reconstruction of the ONH was performed from the B-scan images by MIP rendering using image-processing software (Amira 5.2.2; Visage Imaging, Berlin, Germany) (Fig. 1a). The thin-slab image was then obtained by selecting two planes (approximately 64 μm apart) inside the three-dimensional volumetric image data; only the data within these two planes were displayed in the thin-slab image (Fig. 1b). The LCT was measured as the distance between the anterior and posterior borders at the central three points (100 μm between each point) in each MIP thin-slab image along an axis perpendicular to the anterior LC surface at the measurement point (Fig. 1c). The measurements obtained from the three thin-slab images were used to calculate the mean LCT of the eye.

The LCT was measured using the manual caliper tool of the Amira 5.2.2 software by two observers (EJL and DSL) who were masked to the clinical information for the subject. All measurements were repeated three times each by the two observers, and the average of the six values was used for the main analysis.

### CSF sampling and storage

The CSF was obtained using the fluoroscopy-guided lumbar puncture technique on the same day as the ophthalmic examination [30]. In brief, the participants were positioned in the lateral decubitus position with their neck bent in full flexion and the knees bent in full flexion up to the chest. A standard spinal needle (90 mm length, 20-gauge) was inserted into the subarachnoid space at the L3/L4 or L4/L5 interspace under fluoroscopic guidance. A manometer was connected to a three-way stopcock, and the opening pressure was measured after the column was allowed to equilibrate. CSF sampling was then performed according to a standard protocol [31]. In total, two fractionated CSF samples of 1 and 10–25 ml were collected in separate polypropylene vials. For the macroscopically hemorrhagic CSF samples, additional CSF fractions were collected. All of the CSF examinations were performed by an experienced radiologist (JWL) who was masked to the clinical information for the subject.

The first fraction (1 ml) was used for routine examination of the CSF, which included a cell count and total protein and glucose analysis. Whenever the CSF cell count revealed more than 5 white blood cells or more than 50 red blood cells per mm^3^, the samples were not included in the study (this criterion did not apply to any of the samples). The second fraction (10–25 ml) was used for biomarker analysis. This fraction was centrifuged immediately after sampling (10 minutes at 2000 x *g*), and the supernatant was distributed in different polypropylene vials and frozen in liquid nitrogen. The CSF samples were then stored at −80 °C until analysis.

### CSF analysis

Prior to the CSF analysis, the samples were randomized to obtain a proportional distribution of samples from controls and AD patients on each microtiter plate (40 samples per plate). The laboratory technician was blinded to the expected test outcome in terms of clinical and definitive pathological diagnoses while performing and interpreting the tests.

The CSF levels of amyloid-beta 1–42 peptide (Aβ_1–42_), total tau (T-tau), and tau phosphorylated at threonine 181 (P-tau_181P_) were determined with INNO-BIA AlzBio3 (INNOGENETICS N.V., Gent, Belgium) using Luminex® 200™ (Luminex, Austin, TX, USA). With each assay, the clinical samples together with a blank (sample diluent), the prepared calibrator solutions, and the appropriate controls were tested strictly following the test instructions provided in the kit inserts. All samples were run in duplicate.

If the duplicate results differed by more than 20 % or if the concentrations obtained were out of range, the samples were retested. The concentration ranges of the test kits are described in the package inserts (P-tau_181P_, 9–264 pg/ml; T-tau, 22–1289 pg/ml; and Aβ_1–42_, 56–1710 pg/ml).

### Statistical analysis

To measure the interobserver reproducibility of measurement of the LCT, the intraclass correlation coefficients (ICC) and their 95 % confidence intervals (CIs) were calculated. The Shapiro–Wilk normality test was performed because of the relatively small number of subjects in this study. Comparison between groups for continuous variables was performed using the independent-samples *t* test for parameters that passed normality tests, and the Mann–Whitney *U* test was performed for parameters that did not pass normality tests. For categorical variables, Pearson’s chi-square test and Fisher’s exact test were performed for parametric and nonparametric comparisons, respectively. Linear regression analysis was performed to reveal the factors that influenced the LCT, including age, IOP, central corneal thickness, axial length, CSF pressure, and CSF proteins (Aβ_1–42_, T-tau, and P-tau_181P_), first with a univariate model and then with a multivariate model that included variables from the univariate model for which *P* <0.10. Statistical analyses were performed with Statistical Package for the Social Sciences software for Windows version 17.0 (SPSS, Inc., Chicago, IL, USA). For the factors that showed a significant relationship with the LCT, fractional polynomial analysis [32] was performed using STATA (version 10.0; StataCorp, College Station, TX, USA) to investigate whether the relationship could be better explained by a nonlinear model. The level of statistical significance was set at *P* <0.05.

## Results

The study initially included 52 subjects (22 AD patients and 30 healthy subjects). Of these, eight subjects were excluded because of a diagnosis of concurrent glaucoma (*n* = 3), a poor B-scan quality that did not allow delineation of the LC borders (*n* = 1), poor cooperation during the SD-OCT examination (*n* = 2), and failure to receive a CSF examination (*n* = 2), yielding a final sample of 18 AD patients and 26 healthy subjects.

The final subject sample (19 men and 25 women) were aged 66.0 ± 8.3 years (mean ± standard deviation) and had a spherical error of 0.5 ± 1.9 D. A comparison of the baseline clinical characteristics between the AD and healthy groups is presented in Table 1. The patients with AD were significantly older (69.7 ± 7.6 vs*.* 63.4 ± 8.0 years, *P* = 0.012) and had a lower total Mini-Mental Status Examination (MMSE) score (19.5 ± 3.7 vs*.* 29.0 ± 1.3, *P* <0.001) than the healthy subjects. There were no significant differences between the AD and healthy groups for any of the other baseline characteristics, including gender, spherical error, central corneal thickness, axial length, baseline RNFL thickness, body mass index, and the presence of diabetes or hypertension (Table 1).

**Table 1:** Subjects’ clinical characteristics

	AD group (*n* = 18)	Healthy group (*n* = 26)	*P* value
Gender (male:female)^a^	7:11	12:14	0.760
Age (years)^b^	69.7 ± 7.6	63.4 ± 8.0	**0.012**
Baseline IOP (mmHg)^c^	11.5 ± 2.8	12.9 ± 2.5	0.262
SE (D)^c^	0.1 ± 2.2	0.7 ± 1.6	0.567
Central corneal thickness (μm)^b^	559.6 ± 30.4	572.1 ± 33.6	0.207
Axial length (mm)^b^	23.6 ± 1.0	23.8 ± 1.0	0.519
RNFL thickness (μm)^b^	102.7 ± 12.1	100.0 ± 12.3	0.474
Total MMSE score^c^	19.5 ± 3.7	29.0 ± 1.3	**<0.001**
BMI (kg/m^2^)^b^	23.9 ± 3.3	23.8 ± 2.4	0.957
Presence of diabetes mellitus^a^	3 (16.7 %)	5 (19.2 %)	1.000
Presence of systemic hypertension^a^	10 (55.6 %)	9 (34.6 %)	0.222

Data are presented as the mean ± standard deviation unless otherwise specified. Statistically significant values are shown in bold

^a^Comparisons were made using Fisher’s exact test

^b^Comparisons were made using an independent-samples *t* test

^c^Comparisons were made using a Mann–Whitney *U* test

*AD* Alzheimer’s disease, *BMI* Body mass index, *IOP* intraocular pressure, *MMSE* Mini-Mental Status Examination, *RNFL* retinal nerve fiber layer, *SE* spherical equivalent

Table 2 presents a comparison of the CSF analysis and the LCT between the AD and healthy groups. The CSF pressure was higher in the healthy group than in the AD group, but the difference was nonsignificant (14.4 ± 2.5 vs*.* 12.8 ± 2.6 mmHg, *P* = 0.062; Table 2). There were significant differences between the groups for all of the CSF proteins analyzed; the AD group had a lower Aβ_1–42_ level (306.52 ± 116.21 vs*.* 463.46 ± 124.69 pg/ml, *P* <0.001), a higher T-tau level (110.04 ± 57.08 vs*.* 62.51 ± 19.73 pg/ml, *P* <0.001), and a higher P-tau_181P_ level (51.66 ± 19.40 vs*.* 28.07 ± 14.14 pg/ml, *P* <0.001) than those of the healthy group (Table 2). The interobserver ICC for LCT measurement was 0.926 (95 % CI, 0.901–0.945). No significant differences were found between the groups in any of the superior, central, inferior, and mean LCT measurements (Table 2).

**Table 2:** Comparison of CSF analysis and LCT measurements between AD and healthy groups

	AD group (*n* = 18)	Healthy group (*n* = 26)	*P* value
CSF analysis			
CSF pressure (mmHg)^a^	12.8 ± 2.6	14.4 ± 2.5	0.062
Aβ_1–42_ (pg/ml)^a^	306.52 ± 116.21	463.46 ± 124.69	**<0.001**
T-tau (pg/ml)^b^	110.04 ± 57.08	62.51 ± 19.73	**<0.001**
P-tau_181P_ (pg/ml)^b^	51.66 ± 19.40	28.07 ± 14.14	**<0.001**
LCT measurements			
Superior LCT (μm)^a^	227.61 ± 42.07	238.81 ± 42.08	0.390
Central LCT (μm)^a^	267.72 ± 31.35	262.65 ± 49.05	0.701
Inferior LCT (μm)^b^	232.00 ± 45.67	242.27 ± 46.60	0.423
Average LCT (μm)^a^	242.46 ± 31.93	247.95 ± 37.55	0.616

Data are presented as the mean ± standard deviation, unless otherwise specified. Statistically significant values are shown in bold

^a^Comparisons were made using an independent-samples *t* test

^b^Comparisons were made using a Mann–Whitney *U* test

*Aβ*_*1–42*_ amyloid β_1–42_, *AD* Alzheimer’s disease, *CSF* cerebrospinal fluid, *LCT* lamina cribrosa thickness, *P-tau*_*181P*_, phosphorylated tau_181P_, *T-tau* total tau

**Fig. 2:**
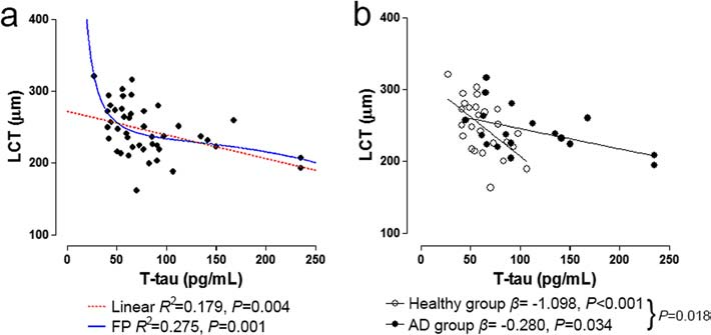
Scatterplots showing the relationship between the cerebrospinal fluid level of total tau (*T-tau*) protein and the lamina cribrosa thickness (*LCT*). **a** Linear regression (*dotted line*) and fractional polynomial (*FP*) analysis (*solid line*) performed across all subjects. **b** Regression analysis with an indicator was performed using the diagnostic group (healthy vs*.* Alzheimer’s disease (*AD*) groups) as an indicator

Table 3 presents the factors associated with the LCT. The univariate analysis revealed a significant influence of higher levels of CSF T-tau (*P* = 0.004) and CSF P-tau_181P_ (*P* = 0.027) and a thinner central corneal thickness (*P* = 0.032) on thinner LCT measurements. In the multivariate analysis, only the CSF T-tau level (*P* = 0.041) was significantly associated with the LCT (Table 3). The relationship was significant for both a linear model (*R*^2^ = 0.179, *P* = 0.004; Fig. 2a, dotted line) and a nonlinear model (*R*^2^ = 0.275, *P* = 0.001; Fig. 2a, solid line). The linear model explained the relationship between the CSF T-tau and the LCT as the following:

**Table 3:** Factors associated with the lamina cribrosa thickness

	Univariate analysis			Multivariate analysis			VIF
	β	95 % CI	*P* value	β	95 % CI	*P* value	
Gender, *male*	5.769	–15.954, 27.493	0.595				
Age, *per 1 year older*	–1.103	–2.371, 0.165	0.087	–0.572	–1.746, 0.601	0.330	1.086
Aβ_1–42_, *per 1 pg/ml larger*	–0.024	–0.100, 0.052	0.521				
T-tau, *per 1 pg/ml larger*	–0.326	–0.543, –0.108	**0.004**	–0.362	–0.709, –0.016	**0.041**	2.843
P-tau_181P_ *, per 1 pg/ml larger*	–0.582	–1.095, –0.069	**0.027**	0.166	–0.627, 0.958	0.674	2.882
CSF pressure, *per 1 mmHg higher*	0.593	–3.526, 4.712	0.773				
Total MMSE score, *per 1 point higher*	0.894	–1.108, 2.896	0.373				
IOP, *per 1 mmHg higher*	0.294	–3.737, 4.325	0.884				
Spherical error, *per 1 D larger*	0.024	–5.983, 6.032	0.994				
Global RNFL thickness, *per 1 μm thicker*	0.930	–0.025, 1.886	0.056	0.845	–0.020, 1.709	0.055	1.113
Central corneal thickness, *per 1 μm thicker*	0.349	0.032, 0.666	**0.032**	0.216	–0.091, 0.524	0.163	1.145
Axial length, *per 1 mm longer*	3.516	–7.861, 14.893	0.536				

Statistically significant values are shown in bold

*Aβ*_*1–42*_ amyloid β_1–42_, *CI* confidence interval, *CSF* cerebrospinal fluid, *IOP* intraocular pressure, *MMSE* Mini-Mental Status Examination, *P-tau*_*181P*_ phosphorylated tau_181P,_ *RNFL* retinal nerve fiber layer, *T-tau* total tau, *VIF* variance inflation factor

$$ y=272.385-0.3255*x $$

The best-fitting nonlinear model described the relationship as the following:

$$ y=229.9+6849*{x}^{-2}-0.000002*{x}^3 $$

where *y* is the LCT measured in micrometers and *x* is the level of CSF T-tau measured in pictograms per milliliter.

Regression analysis with an indicator [33] was performed using the diagnostic group (healthy and AD groups) as an indicator variable. This analysis was performed using the R statistical language version 2.15.1 (http://www.R-project.org (accessed 3 December 2014)). This analysis showed that the association between CSF T-tau protein and the LCT was significant in both the healthy and AD groups, with a stronger association found in the healthy group (regression coefficients = –1.098 vs*.* –0.280, *P* = 0.018; Fig. 2b).

### Representative cases

**Fig. 3:**
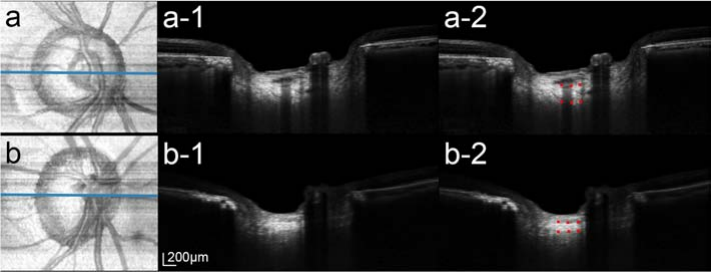
Representative images of eyes from the healthy group **a** and the AD group **b. a**, **b** Enface view of the MIP volumetric image. *Blue lines* indicate the location where the central thin slab MIP images were obtained. **a-1**, **b-1** Thin-slab MIP images obtained at the center of the ONH (*blue lines*). **a-2**, **b-2** Same images as in **a-1** and **b-1** with labels. *Red glyphs* indicate the anterior and posterior borders of the LC. The CSF level of T-tau protein was 27.4 pg/ml **a** and 235.2 pg/ml **b**. Note that the LC is notably thinner in **b**, where the CSF T-tau level was increased

Figure 3 shows representative cases illustrating the relationship between the CSF level of T-tau and the LCT. The LCT is notably thinner in the eye from the AD group, in which the T-tau protein level in the CSF was 235.2 pg/ml (Fig. 3b), than in the eye from the healthy group, in which the T-tau level was 27.4 pg/ml (Fig. 3a).

## Discussion

The LC comprises connective tissue plates that mainly include extracellular matrix (ECM) (i.e., collagen fibers, elastic fibers, basement membranes, and proteoglycans) and a smaller subset of cellular components (i.e., LC cells, astrocytes, and microglial cells). The LC is thought to provide structural and metabolic support to the RGC axons passing through it.

It has been suggested that eyes with thin LC are more susceptible to mechanical damage of the RGC axons. Thinning of the LC may expose the axon to a steep transition between the IOP and the retrolaminar tissue pressure (translaminar pressure gradient) and significantly influence the axoplasmic flow [22, 34–36], causing further axonal damage. However, it is not fully understood whether some eyes have inherently thin LC, or the LC is thinned in the process of degeneration such as physiologic aging or pathologic neurodegeneration such as glaucoma.

The present study suggests a potential mechanism of LC thinning associated with tau pathology; a higher level of CSF tau was associated with a thinner LC both in healthy individuals and in patients with AD. To the best of the authors’ knowledge, this is the first study to reveal a relationship between CSF proteins and the LC characteristics.

Whether CSF tau is a causal factor for thinning of the LC or a consequence of LC thinning is unclear. Several possibilities may be considered for this issue. First, it is possible that the CSF tau is a causal factor in LC thinning. The cytoplasm of astrocytes contains networks of filamentous proteins, such as the actin cytoskeleton, intermediate filaments, and microtubules that maintain tissue and cell integrity, cell mobility, and cell differentiation [37]. It has been suggested that the accumulation of tau contributes to the collapse of the cytoskeleton and degeneration of astrocytes [38]. When increased CSF tau influences the stability of the cytoskeleton of laminar astrocytes, the astrocytes may become more susceptible to degradation, leading to further thinning of the LC. Second, elevated CSF tau may be a result caused by a neurodegenerative cascade facilitated by the thin LC. A thinner LC is associated with a steeper translaminar pressure gradient that causes the axons passing through the LC to experience an abrupt transition between the high IOP and low retrolaminar pressure. Under these conditions, axonal flow can be interrupted [34–36], and this effect may result in the dysregulation of neurotrophic factors [39, 40], which may in turn increase abnormal tau [41–43]. Finally, however, it is also possible that LC thinning is simply an epiphenomenon accompanied by an elevation in CSF tau associated with neurodegenerative processes. The result of this study does not fully explain the causal relationship between the LCT and CSF tau protein; a long-term investigation of larger samples is required to address this issue.

In the present study, more than half of the subjects were healthy individuals; thus, it is not known whether the non-AD subjects with a thin LC or an elevated CSF tau had subclinical AD. Although data were not presented because of the small number of samples, a subgroup analysis performed within the healthy group revealed a marginal but not significant linear association of a lower total MMSE score with thinner LCT (*β* = 10.417, *P* = 0.061). This may indicate that the LCT could also serve as a potential candidate marker representing cognitive function as well as the CSF tau level. A long-term follow-up study is required to verify whether these subjects are more susceptible to developing AD. Additionally, a study that includes subjects with mild cognitive impairment may help to answer this question.

Interestingly, the relationship between the level of CSF tau and the LCT was significant both in the healthy and AD groups, with a stronger relationship found in the healthy group. Although we do not have a clear explanation for this differential correlation between groups, the smaller span of LCT change relative to that of CSF tau level in AD patients might be because of the increased stiffness of the LC tissue in this group, which is associated with older age [44–46]. On the other hand, a relatively larger span of LCT change despite the smaller span of CSF tau level in healthy subjects may indicate that the LCT can serve as a useful marker to monitor subclinical CSF tau increases.

Thus far, the pathogenic influence of tau has mainly been centered on the degeneration of axonal tissue. If this assumption also holds true for RGC degeneration, the CSF tau level should be associated with the number of RGC axons, which is represented by the RNFL thickness. However, in the present study, increased tau was associated only with a smaller LCT and not with a thinner RNFL (data not presented). Instead, a smaller LCT was correlated with both higher CSF tau levels and a thinner RNFL. This finding suggests that CSF tau is more likely associated with glial tissues (LC) than with neural tissues (RGC axons), and the change in the neural tissues might be secondary to that in the glial tissues. This finding supports the assumption that glial cells are more directly associated with neurodegeneration than are neuronal cells [47–49].

In the present study, only the T-tau, not the P-tau_181P_, was associated with the LCT. Although we do not have a clear reason for this result, it is possible that the level of P-tau_181P_ was not sufficiently high to produce a statistical significance. We speculate that other forms of tau phosphorylation could be more highly associated with the LCT. However, the original study (Study for the Usefulness and Standardization of Cerebrospinal Fluid and Plasma Amyloid Biomarkers in Alzheimer’s Disease) did not analyze other types of CSF proteins besides Aβ_1–42_ and P-tau_181P_. Further study is expected to reveal the precise relationship between the phosphorylated tau and LC characteristics.

In the present study, the Aβ_1–42_ level was not associated with the LCT. It is acknowledged that the concentration of Aβ_1–42_ in the CSF increases initially but then decreases decades before the expected onset of symptoms [50–52], whereas the concentrations of CSF tau are increased several years before symptoms develop [50–52] and further increase with the disease progression [53, 54]. In this context, the nonassociation between the CSF Aβ_1–42_ and the LCT and the association between CSF tau and the LCT may suggest that the LCT could serve as a potential clinical indicator for neurodegeneration in patients with AD or those at risk of developing AD.

This study is limited by the small sample size and its cross-sectional design. Hence, a causal relationship between CSF tau and the LCT and the pathogenic significance of this potential relationship could not be determined. A long-term follow-up study with a larger number of subjects, including subjects with RGC pathology, such as glaucoma, is needed to reinforce the findings of this study.

## Conclusions

The present study found that an increased CSF tau level is associated with a thin LC in healthy subjects and in patients with AD. Given the implications of LC thinning in RGC axonal degeneration, it is possible that tau protein contributes to a common pathogenesis of neurodegeneration in the brain and optic nerve. In addition, the LCT could serve as a non-invasive potential biomarker for estimating the level of CSF tau protein and for suspecting the presence of tauopathies. Although the study leaves many questions, its results may provide a better understanding of the mechanisms of tauopathy in neurodegenerative diseases and a basis for future studies investigating the pathogenesis of neurodegeneration in the brain and optic nerve.

## Abbreviations

AD: Alzheimer’s disease
Aβ_1–42_: Amyloid β_1–42_
CI: Confidence interval
CSF: Cerebrospinal fluid
D: Diopters
ECM: Extracellular matrix
EDI: Enhanced depth imaging
ICC: Intraclass correlation coefficient
IOP: Intraocular pressure
LC: Lamina cribrosa
LCT: Lamina cribrosa thickness
MIP: Maximum intensity projection
MMSE: Mini-Mental Status Examination
ONH: Optic nerve head
P-tau_181P_: Phosphorylated tau_181P_
RGC: Retinal ganglion cell
RNFL: Retinal nerve fiber layer
SD-OCT: Spectral-domain optical coherence tomography
T-tau: Total tau
